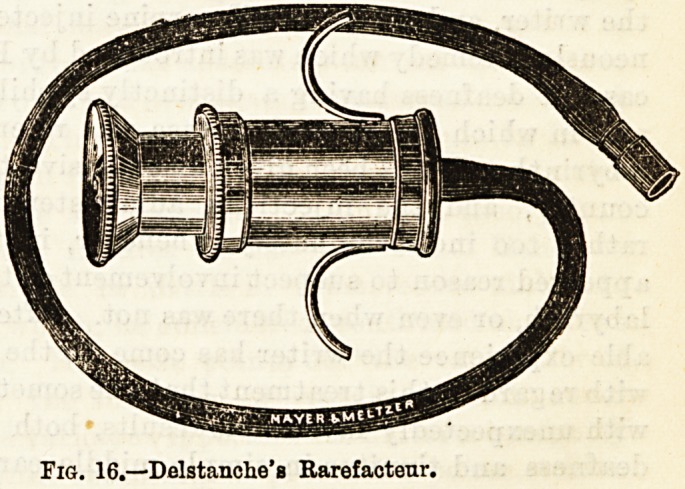# Chronic Catarrh of the Middle Ear

**Published:** 1894-11-24

**Authors:** 


### Medical Progress and Hospital Clinics.

[*The Editor will be glad to receive offers of co-operation and contributions from members of the profession. All letters should be addressed to* The Editor, The Lodge, Porchester Square, London, W.]

### CHRONIC CATARRH OF THE MIDDLE EAR.

### VI.—Treatment.

(*Concluded from page* 117.)

When seromucous secretion is present in the cavity of the tympanum, a series of inflations with Politzer's bag or the catheter is often sufficient to disperse it. If unsuccessful, it becomes necessary to puncture the membrane, and politzerize, by which means the fluid is driven through the perforation. In middle ear catarrh of long standing, in which we find no evidence of fluid in the tympanum, and improvement on inflation is slight, the use of Politzer's method or the catheter should be persevered with until no further benefit is obtained.

The patient can be taught the application of Politzer's bag for himself, but he must be cautioned not to apply the air douche oftener than once a day, as the drum membrane suffers from too frequent distension. The Valsalvian experiment may prove equally injurious if too much resorted to, not for the above reason only, but also on account of the venous con-

Fig. 15.—Dundas Grant's Self-Inflator.

Fig. 16.—Delstanche's Rarefacteur.

gestion of various structures occasioned by it. The temptation to the patient to have frequent recourse to inflation is considerable, owing to the temporary improvement in hearing accompanying increased tension of a relaxed membrane. Dundas Grant has designed an "auto-inflator" for home use which is efficient and inexpensive. (See Fig. 15.) Patients at the Central Throat Hospital are supplied with one of these instruments at a cost of one shilling. The strength of the air current can, of course, be regulated at will, and its passage through the Eustachian tube is greatly facilitated by placing a few drops of the following combination in the receptacle containing the cotton wool, which forms part of the mouthpiece. ℞ Chloroformi ʒiv, ether acetic ʒii, spt. vin. rect. ad. ℥i. Exhaustion of the air in the meatus by Delstanche's rarefacteur (Fig. 16) produces the same effects as already described when speaking of Siegle's speculum, and is very beneficial in some cases; this instrument is also designed for self use.

Complete removal of post-nasal secretion by detergent alkaline lotions, such as Dobell's, and the application of astringent or stimulating remedies to the nasopharynx, the posterior aspect of the soft palate, and around the Eustachian openings should not be omitted. The following are useful examples: ℞ Zinci chlor. gr. xx., glycerini ℥ ss., aq. ad. ℥i. ℞ Menthol ʒ iss., paroleini ℥i. Sig. To be applied with a brush to the posterior wall of the pharynx and naso pharynx night and morning. Medicated vapours may be forced into the Eustachian tube and tympanic cavity in order to promote the solution and absorption of inspissated secretions. Chloride of ammonium generated by means of a Burroughs and Welcome's inhaler is serviceable in suitable cases. Tincture of iodine and acetic ether, in equal parts (20 drops to be added to half-a-pint of hot water and inhaled), is a good combination. Compound tincture of benzoine and oil of pine are also favourite remedies. For the use of these, recourse must be had to "Valsalva." After filling the mouth with the vapour, the nostrils are to be closed tightly, and the cheeks blown out to complete distension. This may be repeated four or five times during five minutes, night and morning. Simple as this method is, it is seldom satisfactorily carried out by patients unless they are specially instructed in its performance. The vapour of chloroform, iodide of ethyl, &c., may be introduced by the surgeon by means of a Politzer's bag fitted with a suitable receptacle, in which is placed some cotton wool holding a small quantity of the drug. Internally we can assist the action of local remedies by the administration of tonics, the most serviceable of which is strychine, combined with iron if anæmia is present.

Disorders of the digestive organs must be set to rights, and special attention paid to the state of the teeth. Chloride of ammonium in twenty-grain doses is beneficial for reducing catarrh, and may be combined with iodide of potash or soda in sclerosis, especially if the patient hears better when he is suffering from a cold. Unfortunately none of these remedies give uniform results, and it may be here stated that treatment of the sclerotic form is very unsatisfactory. Deafness traceable to syphilis is occasionally relieved by large doses of the iodide with or without mercurials, but these specifics do not possess the influence over the disease situated in the middle ear and labyrinth that we are accustomed to see exercised by them over gummatous deposits elsewhere in the body. Tinnitus, as has been already observed, is an unfavourable symptom when accompanied by deafness. By itself it is occasionally a forerunner of sclerosis; in other instances it is traceable to "brain waste," the result of overwork or to mental anxiety, in which case the patient must be encouraged to disregard it, and must be advised to relinquish work for a time and seek change of air and scene. All observers seem to agree that the prospect of relief is brightest when the noises are intermittent. Strychnia may be given as a nerve tonic, and this failing, the bromine salts or hydrobromic acid. Speaking generally, the treatment for tinnitus accompanying middle ear catarrh is identical with that which is directed against the latter; thus iron or arsenic will at once suggest themselves in anæmic states, whereas congestion of the cerebral circulation, if present, must be met by a purgative. There is no doubt that the increased audibility of the subjective sounds on lying down at night is due to altered vascularity, quite independently of the surrounding quietude which, of course, renders them more distinct. A trial is being made at the Central Throat Hospital of small doses of mercury and chalk, gr. $\frac{1}{3}$ t.d.s. (Wingrave), or calomel, gr. $\frac{1}{10}$ (D. Grant). Cases giving evidence of arterial tension are selected, and the results attained have been very encouraging. Some improvement in hearing seems to occasionally attend the diminution of tinnitus. This troublesome symptom has occasionally yielded in the hands of the writer, and others, to pilocarpine injected subcutaneously, a remedy which was introduced by Politzer for cases of deafness having a distinctly syphilitic origin, and in which the seat of disease was referred to the labyrinth. It has been given an extensive trial in this country, and the injections administered, perhaps, rather too indiscriminately, whenever, in fact, there appeared reason to suspect involvement of the nerve or labyrinh, or even when there was not. After considerable experience the writer has come to the conclusion with regard to this treatment that one sometimes meets with unexpectedly favourable results, both as regards deafness and tinnitus in simple middle ear catarrh of not too long standing, and that whenever benefit has accrued from it, the effects have been noticeable by the end of the first week or ten days. After the profuse sudoresis following an injection, the patients express themselves as feeling "clearer in the head," relieved of the "stuffiness," and often, as before observed, of the tinnitus and giddiness. The dose to commence with, is three minims of a 1 in 24 solution, cautiously increasing up to seven or eight minims ($\frac{1}{3}$ gr. pilocarpine). The patient, lying on a sofa, must be well covered with rugs whilst the sweating is going on, and provided with a vessel or a quantity of handkerchiefs in which to void the abundant discharge of saliva. A dose of sal-volatile diminishes the tendency to nausea and faintness. Although the results of the pilocarpine treatment have so far been too uncertain to enable one to give any positive assurance as to the benefit likely to be derived from it, there seems good reason to retain it on our list of remedies in middle ear catarrh. Of its beneficial effects in syphilis of the labyrinth, we have unimpeachable evidence from most competent specialists. Pilocarpine solution may be injected into the tympanum as well as hypodermically, and the combined method is now much adopted. With reference to intra-tympanic injections, it is only necessary to remark that they are easily performed with the aid of the catheter; a few drops of the fluid selected are deposited in the dilated end with a hypodermic syringe or pipette, and propelled into the tympanum by means of an air balloon. The writer does not remember to have seen much permanent benefit follow this practice, though he is aware of at least one patient being rendered worse by it. This is not, however, in accordance with the experience of some writers. A good deal of discrimination is necessary in selecting the fluid to be employed. We should be guided chiefly by the sounds heard through the auscultation tube during catheterisation and by the stage at which the case has arrived. Injections of bicarbonate of soda are indicated when viscid secretions are manifestly occluding the Eustachian tube and cavum tympani. Iodide of potassium in from 1 to 5 per cent. solution is useful later on. Others prefer iodide of ethyl or sterilised vaseline. To this the writer has for some time been in the habit of adding 5 per cent. of menthol with occasional benefit. A combination of menthol and camphor, 1 gr. of each to the ounce of paroleine, is now being tried.

Stricture of the Eustachian tube is capable of being dilated by means of bougies, which are cautiously introduced through the catheter. They are pushed gently forward into the constricted portion and allowed to remain there some minutes; nothing is gained by allowing them to enter the tympanic cavity. The delicate mucous membrane is easily injured by ths bougie, and inflation must not be practised immediately afterwards, for fear of setting up emphysema. The bougies are made of celluloid, in several sizes, and the length of the catheter should be marked in ink previous to introduction.

In treating vertigo, which constitutes the third important symptom of chronic middle ear catarrh, and indicates increase of labyrinthine tension, the cause—which in this form of ear disease arises presumably from structural alterations in the tympanic cavity—must be sought for. The reader need scarcely be reminded that giddiness associated with chronic deafness must be carefully distinguished from what are known as "Menière's Symptoms," dependent on effusion into the semicircular canals, or other labyrinthine change. These symptoms make their appearance suddenly, in a previously healthy adult, and take the form of deafness and tinnitus usually confined to one side, accompanied by giddiness and staggering with a tendency to fall towards the side affected, and followed by nausea and vomiting. There is occasionally loss of consciousness Common aural vertigo may sometimes be relieved by rarefaction of the air in the meatus, especially if the membrane is highly concave. Politzer has divided cicatricial adhesions and a tense posterior fold with benefit. As in dyspeptic vertigo, to which the aural form is, in a sense, related (for it can be induced by errors in diet), internal medication on general principles must not be neglected. Bromine salts are the most useful drugs, preceded for a few days in suitable cases by quinine cautiously administered. Pilocarpine injections may also be tried.

With regard to the operative treatment of middle ear catarrh it may be instructive to enumerate the surgical procedures that have been suggested and practised for its relief, as given by Politzer in his last edition—(1) The artificial perforation of the membrana tympani originally performed by Cooper and Himly;

(2) Section of the posterior fold of the membrana tympani; (3) Tenotomy of the tensor tympani and stapedius muscles; (4) Mobilization and extraction of the stapes; (5) Synectomy of the crura of the stapes. (6) Excision of the whole membrana tympani and the extraction of the malleus and incus. The latter operation has been extensively practised by Sexton in America with encouraging results, especially in regard to giddiness and tinnitus. Further observation is necessary, however, in order to judge correctly of the merits of these operations, and only after the lapse of considerable time can it be determined whether an apparent improvement in the symptoms will continue. Sexton urges the importance of not waiting too long before operating, as the prognosis becomes less favourable the longer this is deferred. If, in conclusion, the student or young practitioner who happens to read these papers on middle ear catarrh is disappointed with the present state of aural therapeutics, as here imperfectly presented, he must act upon St. John Roosa's advice, and seek consolation from the assurance that deafness from this cause is not likely to be so common a disease amongst coming generations as it is now, on account of the increased attention that is being paid to its causation and prevention. The writer hopes his readers will help to hasten the advent of this desirable consummation by making the utmost of the opportunities that they will find abundantly occurring in their own practice.

## Figures and Tables

**Fig. 15. f1:**
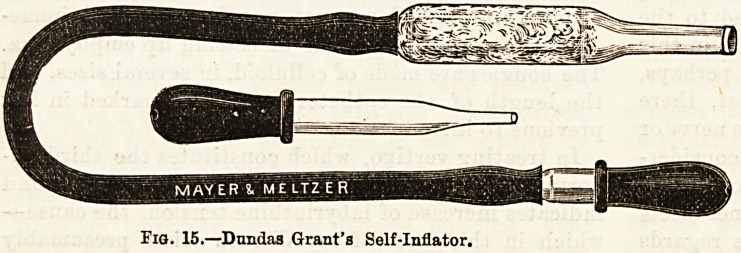


**Fig. 16. f2:**